# Towards the spatial resolution of metalloprotein charge states by detailed modeling of XFEL crystallographic diffraction

**DOI:** 10.1107/S2059798320000418

**Published:** 2020-02-04

**Authors:** Nicholas K. Sauter, Jan Kern, Junko Yano, James M. Holton

**Affiliations:** a Molecular Biophysics and Integrated Bioimaging Division, Lawrence Berkeley National Laboratory, Berkeley, CA 94720, USA; b SSRL, SLAC National Accelerator Laboratory, Menlo Park, CA 94025, USA; c Department of Biochemistry and Biophysics, University of California, San Francisco, San Francisco, CA 94158, USA

**Keywords:** metalloproteins, SPREAD, valence, Bayesian methods, XFEL, spectroscopy

## Abstract

Oxidation states of individual metal atoms within a metalloprotein can be assigned by examining X-ray absorption edges, which shift to higher energy for progressively more positive valence numbers. Indeed, X-ray crystallography is well suited for such a measurement, owing to its ability to spatially resolve the scattering contributions of individual metal atoms that have distinct electronic environments contributing to protein function. However, as the magnitude of the shift is quite small, about +2 eV per valence state for iron, it has only been possible to measure the effect when performed with monochromated X-ray sources at synchrotron facilities with energy resolutions in the range 2–3 × 10^−4^ (Δ*E*/*E*). This paper tests whether X-ray free-electron laser (XFEL) pulses, which have a broader bandpass (Δ*E*/*E* = 3 × 10^−3^) when used without a monochromator, might also be useful for such studies. The program *nanoBragg* is used to simulate serial femtosecond crystallography (SFX) diffraction images with sufficient granularity to model the XFEL spectrum, the crystal mosaicity and the wavelength-dependent anomalous scattering factors contributed by two differently charged iron centers in the 110-amino-acid protein, ferredoxin. Bayesian methods are then used to deduce, from the simulated data, the most likely X-ray absorption curves for each metal atom in the protein, which agree well with the curves chosen for the simulation. The data analysis relies critically on the ability to measure the incident spectrum for each pulse, and also on the *nanoBragg* simulator to predict the size, shape and intensity profile of Bragg spots based on an underlying physical model that includes the absorption curves, which are then modified to produce the best agreement with the simulated data. This inference methodology potentially enables the use of SFX diffraction for the study of metalloenzyme mechanisms and, in general, offers a more detailed approach to Bragg spot data reduction.

## Abstract 2

Electronic configurations at distinct metal centers within a metalloprotein may be characterized by inspecting the scattering factors at the X-ray absorption edge. Such experiments may be feasible at XFEL sources, using Bayesian data analysis.

## 1. Introduction   

**Figure 1:**
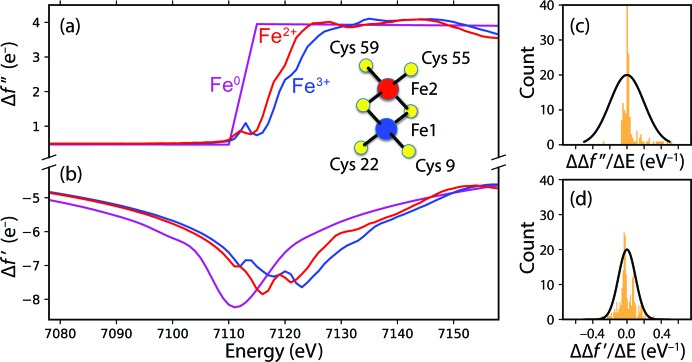
Energy-dependent anomalous corrections to the scattering factor for different valence states of iron. (*a*) Δ*f*′′ correction (proportional to the X-ray absorption) with the near-edge region detected by X-ray fluorescence of Fe^2+^ or Fe^3+^ rubredoxin, courtesy of Darren Sherrell and Graham George (Sherrell, 2014 ▸); and neutral–metal Fe^0^ values taken from the Henke tables as accessed through the *CCTBX* toolbox (Grosse-Kunstleve *et al.*, 2002 ▸). (*b*) Δ*f*′ dispersive correction, related to Δ*f*′′ through the Kramers–Kronig transformation (Smith *et al.*, 2001 ▸). Inset: valence state assignment of the two Fe sites in the [2Fe:2S] cluster of reduced ferredoxin (Einsle *et al.*, 2007 ▸). (*c*) Distribution of ΔΔ*f*′′/Δ*E* when considered in 1 eV increments of *E* over the domain 7070–7170 eV, including both the Fe^2+^ and the Fe^3+^ curves (orange). Based on this, the parameter optimizations presented in this paper assume a prior probability *P*(ΔΔ*f*′′/Δ*E*) = Normal (μ = 0, σ_2_ = 0.2 eV^−1^) to enforce smoothness (black). (*d*) Distribution of ΔΔ*f*′/Δ*E* (orange). The parameter optimizations assume *P*(ΔΔ*f*′/Δ*E*) = Normal (μ = 0, σ_1_ = 0.1 eV^−1^) (black).

For proteins containing transition metal sites, a complete understanding of function requires not only the atomic structure, but also the electronic structure and chemical environment of the metal atoms (Kern *et al.*, 2015 ▸). X-ray absorption spectroscopy has been highly informative, with the extended X-ray absorption fine structure (EXAFS) offering a sensitive measurement of metal–metal and metal–ligand distances, whereas the X-ray absorption near-edge structure (XANES) classically reveals the oxidation state and coordination geometry (Yano *et al.*, 2005 ▸; Glatzel & Bergmann, 2005 ▸). Fundamentally, the *K*-absorption edge, corresponding to the removal of a core 1*s* electron, is shifted to a slightly higher energy when a transition metal is oxidized, as the loss of a valence electron increases the interaction between core electrons and the nucleus (Fig. 1 ▸; Sherrell, 2014 ▸).

Although these absorption-edge methods have been successful, the usual approach of detecting absorption curves by X-ray fluorescence makes it difficult to interpret spectra from metalloprotein systems that have multiple copies of a given metal, due to spectral overlap. An alternative that can distinguish distinct metal centers is to detect the absorption edge through crystallographic diffraction, which inherently provides spatial resolution. In this approach, 3D diffraction datasets are collected from protein crystal(s) using a series of monochromatic energies that span the *K*-absorption edge of the metal in question. Absorption is then quantified by refining wavelength-dependent anomalous correction parameters for each metal. Such data have revealed which of two Fe atoms acts as the electron carrier in the [2Fe:2S] cluster of ferredoxin (Einsle *et al.*, 2007 ▸), and have been used to characterize the mononuclear Fe binding site and the [Mo:7Fe:9S:C] cofactor of nitro­genase (Zhang *et al.*, 2013 ▸; Spatzal *et al.*, 2016 ▸). While macromolecular crystallography is commonly thought of as a technique to determine atomic coordinates, these results show that the absorption edge can readily ascertain the location of a single electron. Spatially resolved anomalous dispersion (SPREAD) potentially offers an independent check on the assignment of heteroatom valence states based on bond distances, such as those assigned by Suga *et al.* (2015 ▸) for the four manganese ions in the [4Mn:5O:Ca] oxygen-evolving complex of photosystem II. It may potentially give a more nuanced view for systems where charge is shared among several metal atoms.

Although the ferredoxin and nitro­genase studies were performed on cryopreserved crystals, it is now widely recognized that a macromolecular structure consists of an ensemble of conformations (Woldeyes *et al.*, 2014 ▸), with crystallography contributing the most relevant information about biological function when the experiment is performed at physiological or room temperature (Keedy *et al.*, 2014 ▸, 2015 ▸; Russi *et al.*, 2017 ▸; Thomaston *et al.*, 2017 ▸). However, dispensing with cryo­preservation presents a general challenge, as it is the principal method used to protect against radiation damage (Garman & Weik, 2019 ▸). Also, with respect to probing the electronic environment, X-ray crystallography studies are particularly difficult for metalloproteins, as metal centers are photoreduced at very low X-ray doses (Yano *et al.*, 2005 ▸; Denisov *et al.*, 2007 ▸; Borshchevskiy *et al.*, 2014 ▸). X-ray free electron laser (XFEL) sources offer a solution to both problems, as the use of femtosecond pulses enables experiments at ambient temperature by producing diffraction prior to the onset of radiation damage, especially when confined to moderate fluences and pulse durations that are available as standard XFEL configurations^1^ (Alonso-Mori *et al.*, 2012 ▸, 2016 ▸; Kern *et al.*, 2013 ▸; see also Lomb, 2011 ▸; Barty *et al.*, 2012 ▸; Nass *et al.*, 2015 ▸). Furthermore, XFEL serial crystallography (wherein the sample is replaced after each shot) has provided high-resolution time-resolved structures (in the 100 fs–400 µs range) for metalloproteins including photosystem II (Young *et al.*, 2016 ▸; Suga *et al.*, 2017 ▸; Kern *et al.*, 2018 ▸), cytochrome c oxidase (Shimada *et al.*, 2017 ▸) and CO myoglobin (Barends *et al.*, 2015 ▸); shot-to-shot X-ray emission spectroscopy can also be used to rule out the presence of unwanted photoreduction (Fuller *et al.*, 2017 ▸; Kern *et al.*, 2018 ▸; Fransson *et al.*, 2018 ▸). All this provides strong motivation to extend the SPREAD method to the XFEL regime, for it would allow detection of time-resolved redox states of complex reaction mechanisms involving multiple transition metal sites.

Realizing this measurement presents profound challenges for both data acquisition and data interpretation. While it is possible to use self-seeding (Amann, 2012 ▸) to produce monochromatic pulses [full width at half-maximum (FWHM) < 1 eV] distributed across the Fe *K*-edge, the use of monochromatic light reduces the number of Bragg spots observed per shot, making it more difficult to acquire complete data with sufficient multiplicity of coverage. Moreover, as the diffraction from each energy channel is observed independently, it becomes difficult to normalize the observations across X-ray wavelengths in order to construct self-consistent absorption curves such as those illustrated in Fig. 1 ▸.

An alternative approach is to take full advantage of the natural bandwidth of the XFEL beam. In principle, since the protein specimen is a crystal, different-energy photons will be split into slightly different diffracted directions, obeying Bragg’s law.^2^ In a similar spirit, protein diffraction data could be collected all at once over a range of X-ray wavelengths, with the results sorted out computationally, using all the data simultaneously to obtain the scattering factors by a global fit. This concept leads us to focus on two experimental features. Firstly, the use of the full self-amplified spontaneous emission (SASE) spectrum of the XFEL source, which has a natural bandwidth on the order of 30 eV when tuned to the metal *K*-edge. This would avoid the loss of fluence that is a consequence of self-seeding, at the cost of mixing the signal from different energies together. However, it is possible to record a detailed image of the stochastically shaped incident spectrum for each pulse (Zhu *et al.*, 2012 ▸), thus providing normalization across energies that can be used to infer statistically the energy-dependent scattering contribution. Secondly, in order to help resolve the energy dependence, the pixel array detector used for imaging the diffraction pattern can be pulled back to a far distance so that Bragg spots are resolved as radial streaks (Bragg’s law dictates that higher energy photons are diffracted to a smaller angle). Even routine XFEL experiments reveal radial streaking that is the result of a combination of energy dispersion and mosaic disorder (Hattne *et al.*, 2014 ▸). In this case, we envision the simultaneous use of two imaging detectors for each diffraction pattern: the ‘right’ side would be imaged by a forward detector to cover as many Bragg spots as possible, and thus determine the orientation of the crystal lattice and the crystal structure by conventional methods, while the ‘left’ side would be imaged further back to resolve the energy-dependent Bragg streaks, with the limitation that the detector would only subtend a few Bragg spots at mid-resolution diffraction angles.^3^ The purpose of this paper is to establish the feasibility of the approach by thoroughly modeling such an experiment. We show through simulation that, given current instrumentation, it is feasible to extract SPREAD spectra.

In the following we attempt to advance the computational methods beyond what has recently been done with XFEL protein crystallography data processing, in several regards. Firstly, due to the need to deconvolute the anomalous scattering factors at different energies, we explicitly model the diffraction as a linear sum over energy-channel contributions. Secondly, since these energy contributions are spread out within each Bragg spot over several pixels, we never explicitly sum the integrated intensity arising from single Bragg spots. Instead, the anomalous scattering parameters of interest are refined directly against individual pixel intensities. Finally, since there is no data reduction step (where pixels within a Bragg spot are summed to a single number), and since the parameter refinement required several tens of thousands of images to converge, we had to implement a parallel computational architecture, where the agreement between model and image data was evaluated over many distributed computer nodes. To provide a context for these developments, we adapted previous software for simulating rotation (Holton *et al.*, 2014 ▸) and still shots (Kirian *et al.*, 2010 ▸) to produce simulated diffraction images that emulate the granular details expected from our proposed data collection strategy. We then used the same tools within a Bayesian framework to analyze the simulated data to produce an accurate maximum likelihood estimate of the energy-dependent absorption from each metal atom.

## 2. Methods   

### 2.1. Parameters of the data simulation   

All data simulations were performed with a *CCTBX* script archived at github (https://github.com/nksauter/LS49/blob/master/sim/step6_batch.py). In the spirit of previous work (Holton *et al.*, 2014 ▸; Holton, 2019 ▸), we attempt to use basic physical principles to derive the diffraction pattern expressed in absolute units (photons pixel^−1^ shot^−1^) so that photon-counting errors may be treated correctly and the experimental feasibility assessed. In addition, the models presented are an attempt to represent the standard configuration of a typical protein crystallography experiment at an XFEL source. This includes the use of a well calibrated, latest generation integrating detector; the delivery of randomly oriented, strongly diffracting crystals by an open-air device such as the drop-on-tape conveyor belt (Fuller *et al.*, 2017 ▸); and the availability of single-shot X-ray spectra that reflect the stochastic nature of the XFEL pulse, as measured in real experimental data. To this end, we treat the experimental parameters as follows.

#### 2.1.1. The imaging detector   

We assume an idealized pixel array detector with a gain of 1.0 (one count per photon), consisting of 3000 × 3000 square pixels of size 0.11 mm, situated 141.7 mm from the crystal, which ensures that the inscribed circle captures the diffraction pattern to an outer resolution of 2.1 Å at the Fe *K*-edge of 7122 eV. It is intended that the analysis of Bragg data in the 2.1–2.5 Å range will allow us to distinguish between the two Fe atoms in the [2Fe:2S] cluster of ferredoxin that are 2.73 Å apart. We assume there is no parallax effect for the detector (Winter *et al.*, 2018 ▸) nor any charge sharing as observed for real pixel arrays (Philipp *et al.*, 2011 ▸). We assume the detector has 1% calibration noise (systematic pixel-to-pixel variation that is constant for a given pixel across repeat simulations due to factors such as impurities in the silicon or differing amplifier settings), but no readout noise (random noise due to pixel electronics).

#### 2.1.2. Simulation of the structure factors   

Structure factors were derived from PDB entry 1m2a (Yeh *et al.*, 2002 ▸), ferredoxin from *Aquifex aeolicus*, in space group *C*2 with unit-cell parameters *a* = 67.2, *b* = 59.8, *c* = 47.2 Å, β = 113.2°. This paper deals with two types of structure factors: the ground truth values, **F**_true_, which are fed into the program *nanoBragg* to produce simulated diffraction images, and the fitted values, **F**_sim_, deduced from the simulated images by computational processing. We use the generic term **F**_model_ to describe either quantity. Operationally, we use the *CCTBX* toolbox to calculate the complex structure factor **F**_model_(λ) for Miller index **h**_0_ at wavelength λ, as the sum of contributions from the explicit atoms listed in the coordinate file plus the bulk solvent (Afonine *et al.*, 2013 ▸; Jiang & Brünger, 1994 ▸):
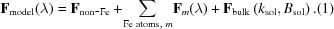
This is the exact procedure used in the program *PHENIX* (Afonine *et al.*, 2012 ▸), with the exception that *k*_sol_ and *B*_sol_, the bulk solvent scale and *B* factors, are set to 0.435 and 46, respectively, in order to minimize the sum over all low resolution (∞–7 Å) amplitudes |**F**_model_(λ)| in accordance with Babinet’s principle. For the present purpose, it is convenient to think of **F**_model_ (λ) as being arranged into two terms:
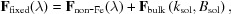
and
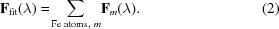
The **F**_fixed_ term includes the scattering from all non-Fe atoms, calculated by the fast Fourier transform method (Ten Eyck, 1977 ▸; Grosse-Kunstleve *et al.*, 2004 ▸). Although the structure does contain other anomalous scatterers such as S and Zn, these anomalous contributions vary only weakly near the central energy of this experiment (7122 eV), so they are evaluated once at that energy and held constant throughout the remainder of the data simulation and analysis. For the diffraction analysis of Section 2.4, **F**_fixed_ is taken to be a known quantity.

The **F**_fit_ term is the sum, over all Fe atoms in the unit cell, of the energy-dependent contribution evaluated by the usual direct-summation formula,
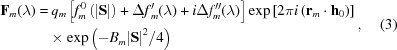
where *q*_*m*_ is the occupancy of metal *m*, |**S**| is the magnitude of the scattering vector (= 1/resolution), **r**_*m*_ is the position vector of the atom expressed in unit cell fractional coordinates, **h**_0_ is the Miller index and *B*_*m*_ is the isotropic *B* factor of the atom. In this expression, *f* ^0^ represents the normal (non-anomalous) scattering factor of the atom, dependent on the scattering vector but not on energy (and we assume negligible dependence on the oxidation state). The Δ*f*′ and Δ*f*′′ terms represent the real and imaginary components of the anomalous scattering that are dependent only on energy and valence state. Ground truth (**F**_true_) for the present data simulation is for the Fe1 atom to be oxidized and the Fe2 atom to be reduced (Einsle *et al.*, 2007 ▸) with corresponding Δ*f*′ and Δ*f*′′ values taken from Sherrell (2014 ▸), see Fig. 1 ▸. In contrast, for the structure factor analysis of simulated images (**F**_sim_), the **F**_fit_(λ) subterm embodies the (initially unknown) wavelength-dependent anomalous structure factors Δ*f*′ and Δ*f*′′ that we endeavor to recover.

#### 2.1.3. Simulation of the mosaic crystal   

**Figure 2:**
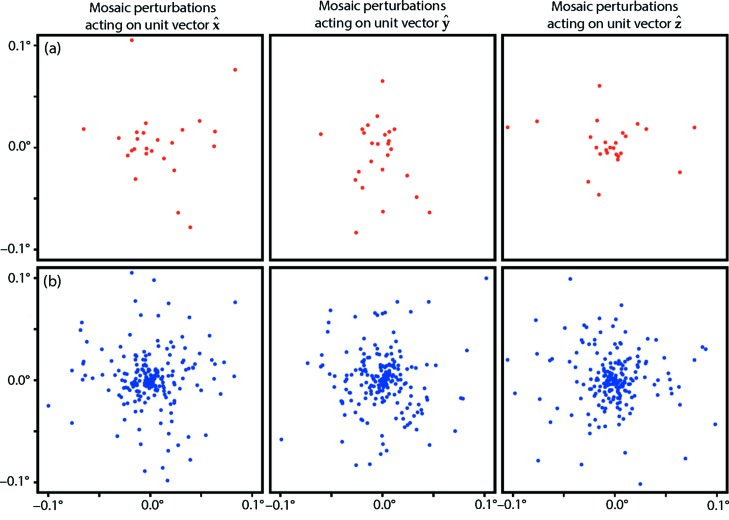
Mosaic rotational model used for (*a*) image simulation and (*b*) data analysis. To create each simulated image, the crystal volume is broken into 25 separately rotated mosaic domains of equal volume, each of which diffracts independently, with the final diffraction representing a sum over all contributions [equation (6)]. Each of the 25 domains has a slightly perturbed orientation with respect to the randomly chosen reference orientation of the crystal as a whole [equation (5)]. (*a*) illustrates the ensemble of these perturbations, plotting the action of the 25 rotation matrices **U**_*D*_ on **x^**, **y^** and **z^** unit vectors attached to the reference crystal, with displacements expressed in degrees, while (*b*) represents the 200 domains used for data analysis. A critical assumption is that the crystal contains a smooth continuum of domain orientations, thus satisfying the Bragg diffraction condition over a range of incident energies. If the number of domains were small (*N*_*D*_ ≪ 25) or the distribution of perturbations non-Gaussian, then it would be difficult to find mutual scaling factors for the diffraction from different energy channels of the SASE pulse. For simplicity, the same ensemble of 25 perturbations **U**_*D*_ was used for all image simulations; however, this did not prevent the simulated data from being successfully analyzed under the assumption of a smooth distribution.

To simulate a diffraction image typical of shots taken at beamlines like the Macromolecular Femtosecond Crystallography (MFX) instrument at LCLS (Boutet *et al.*, 2016 ▸), we assume that a perfectly collimated beam with a 1 µm^2^ focus intersects a 4 µm path through the crystal. Consistent with the practice of Busing & Levy (1967 ▸), we express the crystal orientation in reciprocal space (Sauter *et al.*, 2006 ▸) as the matrix
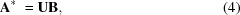
where the reciprocal space orthogonalization matrix **B** represents the reciprocal unit cell basis vectors (a*|b*|c*) arranged in a conventional reference orientation, and **U** is a unitary rotation matrix chosen at random for each shot. However, we also wish to model the mosaic disorder of the crystal (Nave, 1998 ▸). Therefore, we break up the diffracting crystal volume into 25 congruent but separately rotated domains (blocks), with indices *D* = 1, …, 25, each of which contributes independently to the diffraction, thus the structure factor intensities (not the amplitudes) are summed. We derive an effective orientation matrix for each domain,
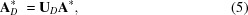
where the 25 **U** *_D_* are rotation matrices with axes randomly chosen from the unit hemisphere and rotational magnitudes drawn from a Gaussian with a standard deviation of η = 0.05° (Fig. 2 ▸). This set of 25 perturbation matrices is generated by the source code at https://github.com/nksauter/LS49/blob/master/tests/tst_mosaic_orientations.py.

#### 2.1.4. Incident X-ray pulses   

**Figure 3:**
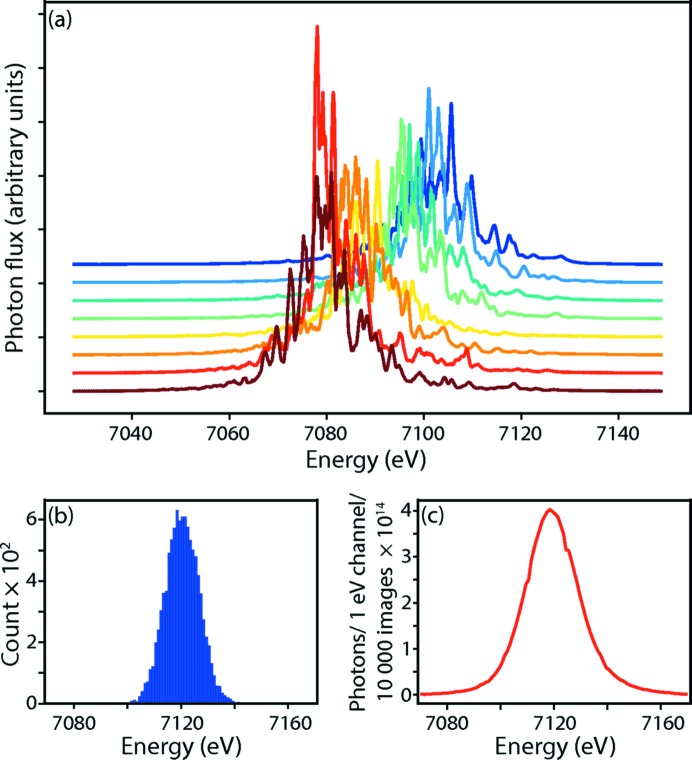
Properties of the incident X-rays. (*a*) Eight randomly chosen LCLS spectra from experiment LG36. Each XFEL pulse has a randomly shaped spectrum with a unique total fluence and mean energy. Each curve is plotted with a separate vertical offset for clarity, but all share the same horizontal scale. (*b*) Distribution of mean pulse energies used for the simulation (over 10 000 pulses), centered at the Fe *K*-edge at 7120 eV with a standard deviation of 6.3 eV. (*c*) Cumulative intensity distribution over 10 000 pulses centered at 7119 eV with a full width at half maximum of 22 eV (0.3% Δ*E*/*E*).

To simulate SASE pulses with properties similar to those at LCLS, we began with actual spectra (Zhu *et al.*, 2012 ▸) measured in the front-end enclosure during a 14 min period (run 209) of LCLS user proposal LG36, centered at 7088 eV (Fig. 3 ▸). Starting with a separate spectrum for each simulation, we applied a baseline correction above and below the energy region of interest, and an FFT-based low-pass filter to smooth out any features narrower than about 1 eV. Furthermore, we translated the energy scale to center the average spectral maximum at the Fe *K*-edge (7120 eV), and defined the intensity scale to give an average integrated number of photons over the entire run of 10^12^ photons shot^–1^. The 7070–7170 eV range was then downsampled into exactly 100 energy channels, thus providing a distribution of stochastic spectral shapes, total fluences and mean energies. We assume that the beam is polarized with the **E**-vector horizontal.

### 2.2. Simulated diffraction   

The kinematic theory (single-scatter from crystals small enough to ignore attenuation) is presented in the classic literature (James, 1962 ▸) and has recently been applied to the simulation of both synchrotron-based rotation images (Diederichs, 2009 ▸; Holton *et al.*, 2014 ▸) and XFEL-based still shots (Kirian *et al.*, 2010 ▸; Kroon-Batenburg *et al.*, 2015 ▸). Given the wavevectors of the scattered (**s**_1_, defined by the pixel position) and incident (**s**_0_) X-rays, both vectors of length 1/λ, and defining the scattering vector as **S** = **s**_1_ − **s**_0_, we compute the crystal diffraction intensity for a single pixel on a femtosecond still shot (photons pixel^−1^) as follows:
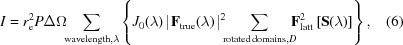
where *r*_e_ is the classical radius of an electron (2.62 × 10^−15^ m), *P* is the polarization factor (Kahn *et al.*, 1982 ▸) in the direction of the pixel and ΔΩ is the solid angle subtended by the pixel in steradians. Within the detailed summation, *J*_0_(λ) is the incident fluence (photons channel^−1^ m^−2^), and **F**_true_ the ground-truth energy-dependent structure factor of the unit cell [equation (1)] taken at the nearest Miller index **h**_0_ to the position of the pixel in reciprocal space. The **F**_latt_ structure factor is the Fourier transform of the finite array of lattice points that make up the crystal or mosaic domain. Like any structure factor, **F**_latt_ is the ratio of the scattered wave from the object of interest to that of a single electron at the origin (Hartree, 1925 ▸). In the case of **F**_latt_, the object is the lattice points themselves and for **F**_true_ or **F**_sim_ it is the contents of one unit cell. **F**_latt_ is multiplied by **F**_true_ because the cell is convoluted with the lattice, and convolution in real space is a product in reciprocal space. At the exact center of each recip­rocal lattice point (RLP), where the Laue conditions are met, **F**_latt_ is equal to the number of unit cells in the mosaic domain, while in the surrounding neighborhood **F**_latt_ takes on a shape essentially identical to the Fourier transform of the average mosaic domain shape. Smaller mosaic domain size therefore leads to larger spots. In the special case where the crystal is a lattice of dimensions *N_a_* × *N_b_* × *N_c_* (unit-cell counts along the *a*, *b* and *c* axes), an exact expression for **F**_latt_ is a 3D version of the grating function, as employed by Kirian *et al.* (2010 ▸). However, in this study we assume a much larger crystal (Section 2.1.3) consisting of many mosaic domains with a distribution of shapes and sizes (Nederlof *et al.*, 2013 ▸), and thus we model the average coherently diffracting volume as a 3D Gaussian. The Fourier transform of this is a Gaussian RLP, which we approximate with the following peak profile:
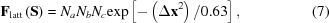
where Δ**x** is the distance to the center of the RLP expressed in units of the reciprocal domain size:
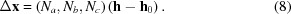
Here, **h** is the real-valued Miller index corresponding to the pixel (or diffracted ray) of interest,
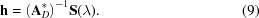
Miller indices are generally expressed as integers, but because every pixel has a location in reciprocal space, it may conveniently be given a non-integer value **h**. The nearest integer-valued Miller index **h**_0_ is the same used in equations (1) to (3) to select an appropriate **F**_model_ for each pixel. The factor 0.63 in equation (7) was chosen to force the RLP volume and FWHM to be similar to that from a rectangular-volume domain. The simulations presented here (expressed in the choice of parameters *N_a_*, *N_b_*, *N_c_*) were equivalent to modeling mosaic domains with an average full width at half-maximum diameter of *D*_eff_ = 400 nm.

**Figure 4:**
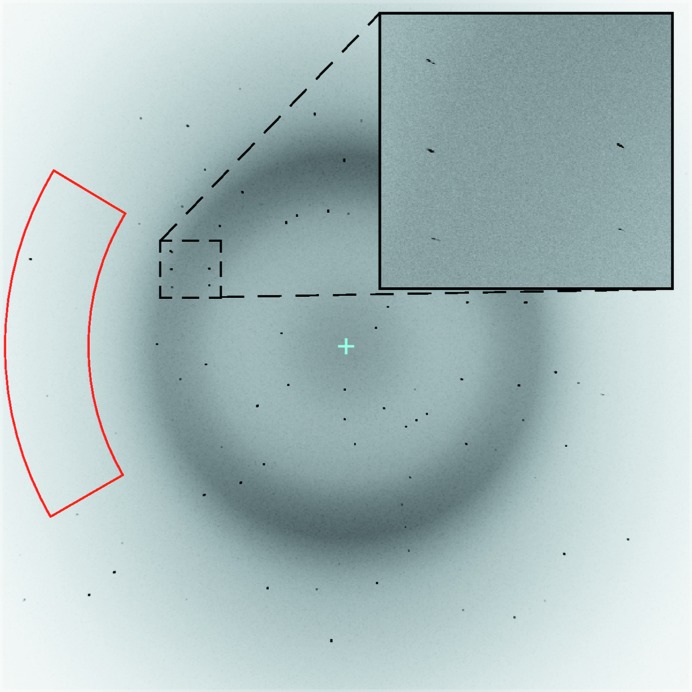
Typical diffraction simulation from a randomly oriented crystal. The detail in the inset confirms that Bragg spots have the appearance of radially oriented streaks, resulting from the combined effects of the broad XFEL bandpass, crystal mosaicity and energy-dependent structure factors. The region of interest (red) defines the subset of data in the 2.1–2.5 Å annulus, and within position angles 150–210°, selected for the analysis of Fe scattering factors. Although the crystal scale factor *G_L_* is generally considered to be resolution-dependent for data merging (Bolotovsky *et al.*, 1998 ▸), the use of a narrow resolution annulus in this case justifies the use of a single constant in equation (16).

In addition to the crystal diffraction, our simulation added the diffraction from the liquid-droplet carrier used for sample delivery (Fuller *et al.*, 2017 ▸), and from the atmospheric path between the crystal and beamstop. Liquid was represented by a 100 µm path through water, and air by a 10 mm path through N_2_, as described in the supplementary materials of Holton *et al.* (2014 ▸). No attempt was made to model the effect of diffuse scattering (Wall *et al.*, 2018 ▸), and the absorption of the X-ray beam in the sample and air was neglected. Once the contributions of crystal, liquid and air were summed, shot noise was added by replacing the expected average photon count Λ_*i*_ of pixel *i* with the value *k_i_* sampled from a Poissonian distribution, which has the probability density function
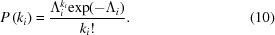
Diffraction simulations were performed with randomly chosen crystal orientations (Fig. 4 ▸). The original standalone *nanoBragg* was refactored into a C++ class and provided with Python bindings within the *simtbx* (simulation toolbox) directory of the *CCTBX* project (Grosse-Kunstleve *et al.*, 2002 ▸). The Python/C++ interface was configured so as to reuse code objects within the *dxtbx* (diffraction experiment toolbox) that provide a physical description of the experiment, including the beam, crystal and detector (Parkhurst *et al.*, 2014 ▸). Parallel execution was achieved at the Python level by delegating diffraction patterns from independent crystals to separate worker ranks with the message passing interface (MPI), while the C++ loop over image pixels was accelerated using several parallel threads to simulate independent pixels with OpenMP. Overall wall-clock calculation times for different high-performance computing systems are shown in Table 1 ▸; pixel values on the three systems were numerically identical provided that the same random number seeds were given. A GPU-accelerated version of *nanoBragg* has also been prototyped (James Holton and Giles Mullen, unpublished work).

### 2.3. Preliminary analysis of the simulated data   

We now switch the point of view, treating the simulated images from Section 2.2 as a real serial crystallography dataset, and ask what data analysis protocols are required to deduce the ferredoxin Fe anomalous corrections Δ*f*′ and Δ*f*′′.

#### 2.3.1. Conventional data reduction   

**Figure 5:**
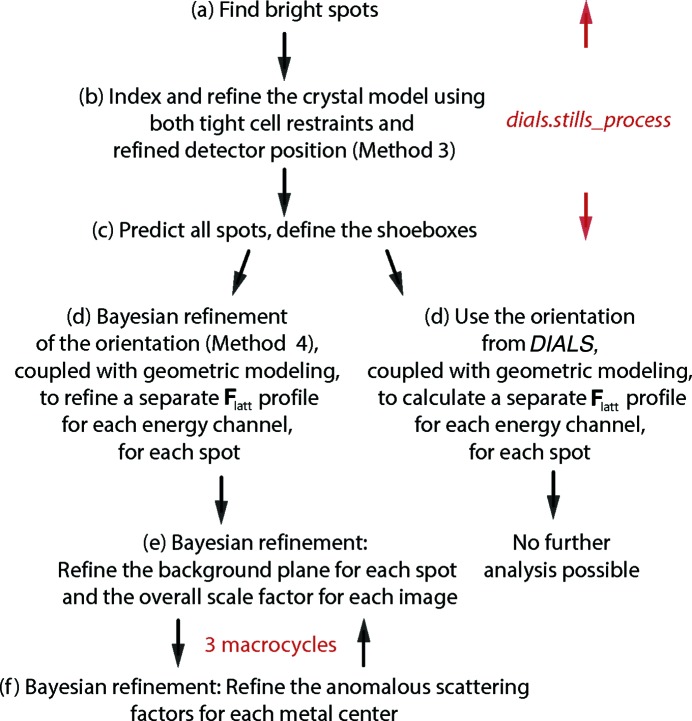
Data analysis protocol. Of 100 000 simulated patterns, 99 979 process correctly with *dials.stills_process*. Exact software parameters and command line scripts for *DIALS* processing (*a*)–(*c*) and *CCTBX* modeling (*d*)–(*f*) are documented in the github repository at https://github.com/nksauter/LS49 under paper1, particularly in the README file.

**Figure 6:**
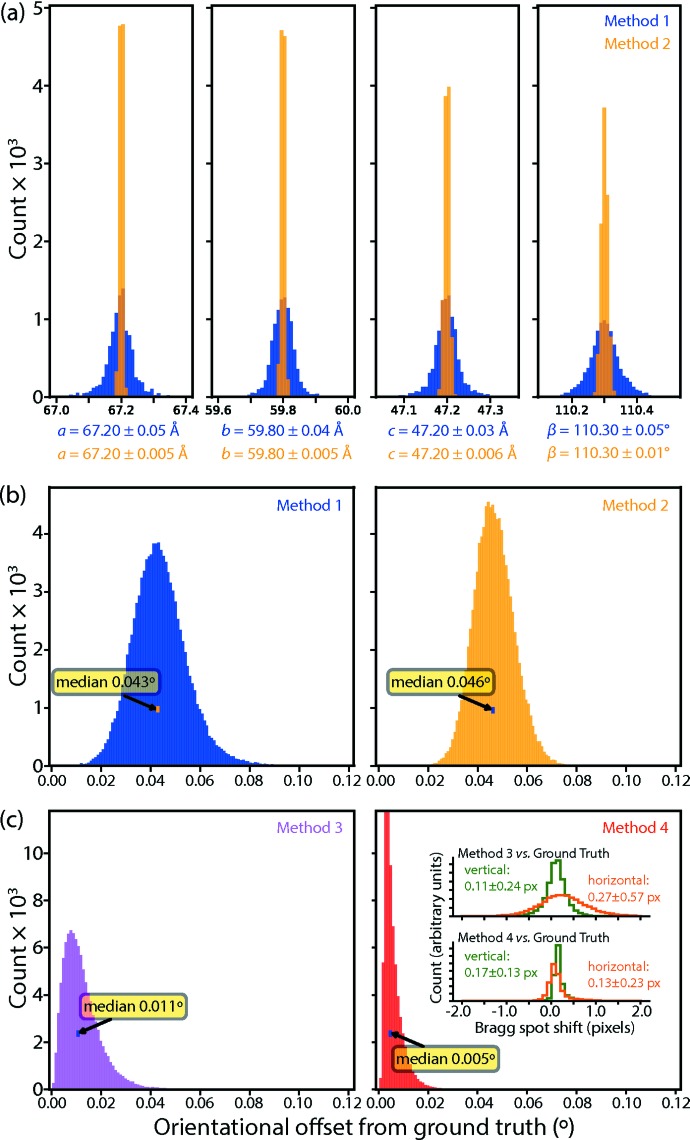
Comparison of refined crystal models against ground truth. (*a*) Distribution of unit-cell parameters. Method 1 (blue), parameters are fit freely with *DIALS* against the bright spot positions; Method 2 (orange), tight restraints are applied for *DIALS* refinement: *a* = 67.2 ± 0.002, *b* = 59.8 ± 0.002, *c* = 47.2 ± 0.002 Å, * β* = 110.3 ± 0.0034°. (*b*) and (*c*) Distribution of angular offsets of the unit-cell basis vectors (averaged over **a**, **b** and **c** for each lattice), in comparison with the ground truth. Specifically, this refers to the ‘fine-grained’ ground truth, which is the average over all 25 A*_D_ matrices shown in Fig. 2 ▸, which is about 0.0077° offset from the ‘coarse-grained’ ground truth that is simply the randomly oriented A* constructed as input to the simulation. In Method 3 (magenta), the corrected detector position (1/2 pixel horizontal and vertical offsets) is provided prior to *DIALS* refinement, and in Method 4 (red), optimized rotational perturbations are applied to align the *nanoBragg*-predicted spot profiles with the ground truth data images (Appendix *B* [App appb]). Insets show the distribution of positional offsets of the Bragg spots in the Fig. 4 ▸ region of interest, comparing either Method 3 or Method 4 with the ground truth.

We began with routine data processing with the program *dials.stills_process* (Brewster, 2016 ▸; Brewster *et al.*, 2018 ▸), yielding estimates of the unit-cell parameters and crystal orientation (Fig. 5 ▸), encapsulated in the 3 × 3 orientation matrix A*. Several attempts were needed before it was ultimately possible to deduce the Fe anomalous scattering corrections (as judged by r.m.s.d. comparison to the ground truth, see Section 3 below). In Method 1, the matrix A* was refined to fit the data without further restraint. The average unit-cell parameters agreed exactly with the ground truth as defined by the PDB file. However the variance levels, with standard deviations of about 0.06% for each parameter [Fig. 6 ▸(*a*), blue traces], were prohibitively large for modeling the scattering factors. We therefore introduced Method 2: the application of isomorphism restraints with the tie_to_target command option of *DIALS*. Here the initially determined unit-cell parameters were used as tightly restrained targets, resulting in very small standard deviations on the order of 0.01% [Fig. 6 ▸(*a*), orange traces]. However, even with these improved unit-cell parameters, the crystal orientations were still misaligned from the ground truth with a median missetting angle of 0.046° [Fig. 6 ▸(*b*)], which proved prohibitively large. The cause turned out to arise from mutually inconsistent definitions of the detector origin between the simulation script (following the *MOSFLM* convention) and the *DIALS* analysis program, amounting to a 1/2 pixel offset in the horizontal and vertical directions (see Holton, 2019 ▸, Section 2.3, paragraph 2).^4^ The corrected detector position was provided back to *dials.stills_process* as a reference for a third round of indexing and crystal orientational refinement (Method 3). This reduced the median missetting angle to 0.011° [Fig. 6 ▸(*c*), magenta], but it was still insufficient for further progress.

#### 2.3.2. Orientational refinement based on spot profiles (Method 4)   

When decomposed into rotational missettings about the horizontal, vertical and X-ray beam axes, the only significant contributions were along the horizontal and vertical axes (data not shown). The path forward became clear by using the Method 3 orientation matrices to create *nanoBragg* image simulations, and noting that the Bragg spot positions in the region of interest (Fig. 4 ▸) were up to one pixel out of position, compared with the corresponding original simulations of Section 2.2. We therefore set up a parameter optimization problem to apply horizontal and vertical rotational perturbations to the lattice model, such that the resulting *nanoBragg* spot simulation would be most consistent with the shape and position of spots on the reference image. As this depends on Bayesian concepts presented below (Section 2.4), the full description of Method 4 is saved for Appendix *B* [App appb]. Fig. 6 ▸(*c*) shows the consequent improvement in spot position, as well as the reduction in the median missetting angle to 0.005°.

### 2.4. A Bayesian approach to modeling the anomalous signal   

We now further examine whether the anomalous scattering curves can be extracted separately for each Fe atom.

Here we make the following assumptions about what is already known. From conventional data reduction (Section 2.3.1), we have complete knowledge of the non-anomalous Bragg spot intensities. Therefore, we can solve and refine the crystal structure, thus producing a coordinate model that permits us to derive **F**_fixed_ from equation (2). For the two metal atoms described by **F**_*m*_ in equation (3), we know coordinates **r**_*m*_ and *B* factors *B*_*m*_; but are still missing Δ*f*′_*m*_(λ) and Δ*f*′′_*m*_(λ). We assume that there is accurate knowledge of the unit-cell parameters, which have a very narrow distribution [*e.g.* Fig. 6 ▸(*a*), Method 2], that the mosaic rotation parameter η and effective mosaic domain size *D*_eff_ are known (Sauter *et al.*, 2014 ▸), that the detector geometry and position are known to high precision (Brewster *et al.*, 2018 ▸), and that the single-shot spectrometer gives an accurate knowledge of the X-ray spectrum *J*_0_(λ) that is incident on the crystal. What is still left to model [in addition to Δ*f*′_*m*_(λ) and Δ*f*′′_*m*_(λ)] is a better estimate of the orientation matrix A* as mentioned above (Section 2.3.1, Method 4), the overall scale factor *G*_*L*_ for each image *L*, and the background photon level *g_x_* behind each Bragg spot *x*.

**Figure 7:**
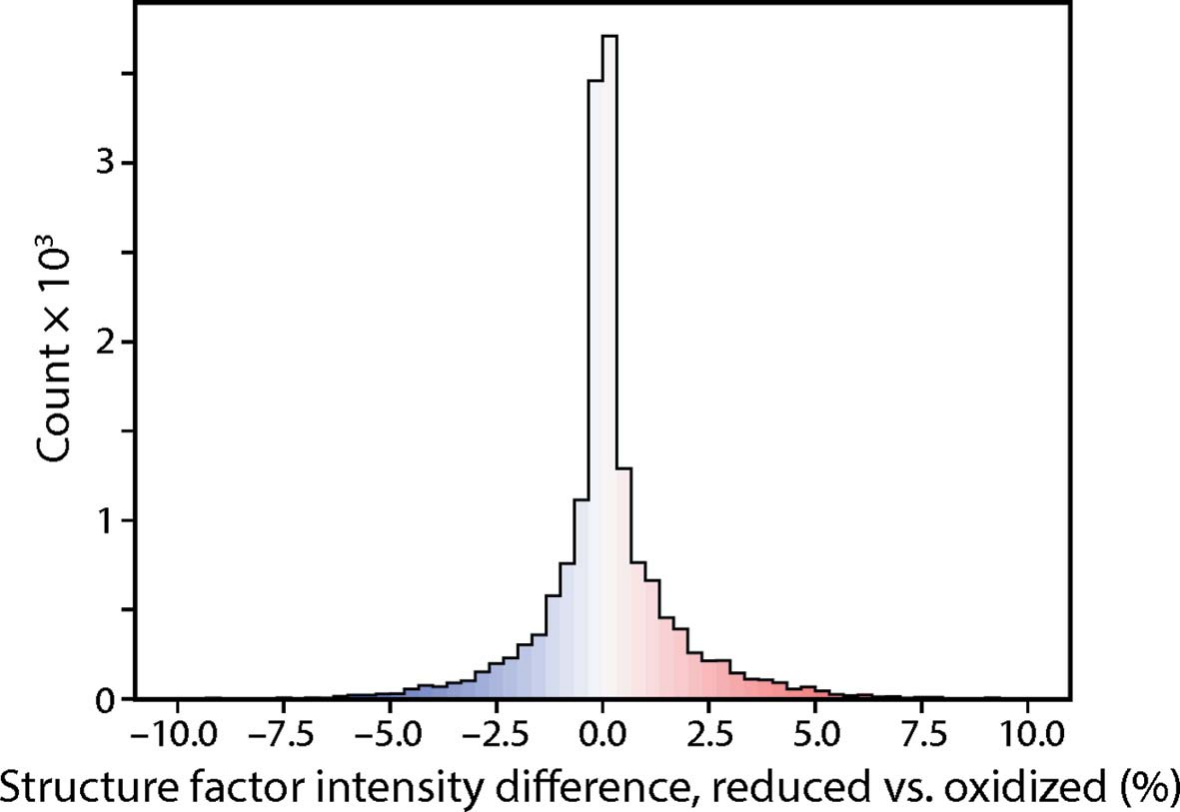
Effect of adding one valence electron on the structure-factor intensities. Starting with the published PDB structure (1m2a), and using the anomalous scattering factors of Fig. 1 ▸, the structure factors (including bulk solvent) are calculated at 7122 eV for the oxidized and reduced forms of ferredoxin, for Miller indices in the 2.1–2.5 Å resolution range. The plot shows the change upon reduction of the structure-factor intensity |**F**_true_(λ = 7122 eV)|^2^ normalized by the average intensity in that range. The r.m.s. difference is 1.7%, sizable enough to permit the modeling of anomalous scattering factors demonstrated in Table 3 ▸. A number of intensities (173 of the total 8 234) change more than 5%.

**Figure 8:**
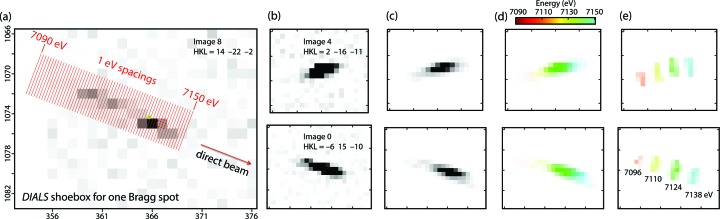
Pixel-level analysis of the Bragg spot observations. (*a*) Detail of one Bragg spot from a simulated image, focusing on the ‘shoebox’ identified by *DIALS* as the bounding box for the signal and surrounding background. Due to Bragg’s law (λ = 2*d*sinθ), pixels at different diffraction angles θ correspond to different X-ray wavelengths, along a line radially extending from the direct beam position. In this instance a 1 pixel dispacement corresponds to a 3.8 eV energy difference, yet the approach of this paper allows us to combine data from many spots to effectively resolve scattering factors at the electron-Volt level. (*b*) Two other simulated Bragg spots, along with (*c*) a model of each spot omitting the background scatter and shot noise. (*d*) As in (*c*), but color coding each pixel by the average X-ray energy represented by the recorded photons, and (*e*) separate calculations of the **F** ^2^_latt_(**S**) factor contributed by four separate energy channels (7096, 7110, 7124 and 7138 eV). (*e*) assumes equal incident photon intensities for each channel.

The usual cautions apply when considering anomalous scattering from a protein, as the signal is weak. However, for a subset of Miller indices (Fig. 7 ▸) the addition of one valence electron to a single Fe atom can change the intensities as much as 5% or more, therefore we expected the desired signal to be embedded in our data. For data analysis, we avoided the routine strategy of integrating the Bragg spots and merging the signal for repeat observations of the same Miller index. Instead, we took full advantage of positioning the detector far back from the crystal, thus allowing the mosaic crystal to act as a spectral analyzer, spreading out the diffracted X-rays over the spectrum of incident energies (Fig. 8 ▸). In an idealized case we would simply read the intensity profile along the energy scale illustrated in Fig. 8 ▸(*a*), but in the present case it is more complicated for several reasons. Firstly, we aim to resolve the anomalous scattering factors Δ*f*′ and Δ*f*′′ with a spacing of 1 eV on the energy axis, while our simulation was intentionally modeled with a challenging 3.8 eV separation per pixel. Therefore, an appreciable amount of deconvolution will be needed. Secondly, each energy channel contributes a different flux *J*_0_(λ) to the diffraction pattern (Fig. 3 ▸); lastly, the effect of crystal mosaicity is to further spread out the diffraction contributed by each wavelength so it is smeared out over several pixels [Fig. 8 ▸(*e*)], as determined by the spot profile factor in equation (6), ∑_*D*_ **F** ^2^_latt_[**S**(λ)]. All these phenomena lead to the necessity of combining all the available information simultaneously, including the structure factors for the protein (except for the unknown anomalous contribution of the Fe atoms), the recorded spectra and the best orientation and mosaicity of all crystals determined from indexing, all in order to estimate the Δ*f*′ and Δ*f*′′ scattering parameters statistically. We take the normal Bayesian approach, which is nicely introduced in its application to crystallography by McCoy (2004 ▸). Bayes’s theorem states that the posterior probability of the model (consisting of our parameter estimates), given the data, is proportional to the likelihood of the data given the model and to the prior probability of the model:
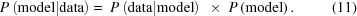
The probability of the data *P*_data_, which normally appears in the denominator of Bayes’s theorem, is constant in our situation and is thus omitted here. *P*(data|model) is assumed to be independent for each pixel, therefore the collective likelihood is the product of individual pixel likelihoods, taken over all pixels *i*, including all Bragg spots observed over all images,
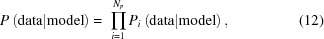
where *N*_p_ is the number of pixels. We will find the most likely model parameters {*z*} by minimizing a loss function that takes the negative log of the posterior probability,
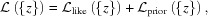
where
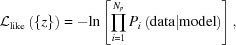
and
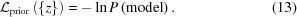
We will use Poissonian statistics [equation (10)] to compute the probability of observing the pixel value *k_i_* given the model value Λ_*i*_. Combining equations (10) and (13) gives
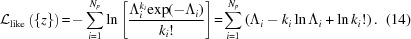
The Poissonian probability is valid as long as the model and data are expressed in units of photons rather than detector pixel units, so there is an implicit assumption that there is a good understanding of the detector gain. The last term involving *k_i_*! is independent of the model parameters, and therefore constant and of no consequence for the parameter fitting, so it is dropped:
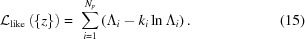



#### 2.4.1. Modeling the pixel’s photon count Λ_*i*_   

We intend for the target function 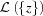 to be summed over all pixels *i* of each rectangular shoebox *x* containing a strong Bragg spot (as identified by *dials.stills_process* and illustrated in Fig. 8 ▸). Therefore the model must cover the contributions of Bragg diffraction as well as the background *g*_*x,i*_ due to liquid and air scatter,
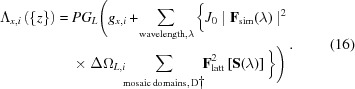
where *G_L_* is a scale factor applied to all shoeboxes on a single image *L* that converts the model value to photons pixel^−1^, compensating for the arbitrary scale of the model.^5^ Note that **F**_sim_(λ) = **F**_fixed_ + **F**_fit_(λ) is the total structure factor recovered from the image data, but the subterm **F**_fixed_ is extracted from **F**_true_ and not allowed to vary. We treat the background *g_x,i_* separately for each Bragg spot observation *x* using a best-fit plane as employed previously (Rossmann, 1979 ▸; Leslie, 1999 ▸),
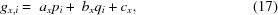
where *p_i_* and *q_i_* are the slow and fast pixel coordinates of the shoebox, respectively. The background scatter is only weakly dependent on wavelength, so we make it independent of *J*_0_(λ) in equation (16). Altogether, the unknown parameters to be determined by maximum-likelihood fitting are the per-image scale factors *G*_*L*_, the per-spot background parameters {*a_x_*,*b_x_*,*c_x_*} and the {Δ*f*′_*m*_(λ), *f*′′_*m*_(λ)} scattering factors for metals Fe1 and Fe2 over 100 energy channels [that determine **F**_fit_(λ)].

As for the geometric spot profile ΔΩ_*L,i*_∑_*D*†_ **F** ^2^_latt_[**S**(λ)], it has dependence primarily on the crystal orientation A* determined by *DIALS* or by profile-based orientational refinement; therefore it can be precalculated in a separate step [one value for each energy channel; Fig. 5 ▸(*d*)]. For this purpose we used a large (200 member) ensemble *D* ^†^ to adequately sample the rotational mosaicity [Fig. 2 ▸(*b*)] rather than the small sample [Fig. 2 ▸(*a*)] used for the simulation. We used the ground truth mosaic rotation (0.05°) and domain size (400 nm) for the present calculation, but assume that in real cases these values can be experimentally determined as in the work by Sauter *et al.* (2014 ▸).

#### 2.4.2. Restraints   

While the parameters {Δ*f*′_*m*_(λ), *f*′′_*m*_(λ)} should be overdetermined by the data, there is still considerable noise, as well as poor energy coverage far away from the 7120 eV set point [Fig. 3 ▸(*c*)]. Thus, there is a danger that the parameter estimates may diverge during the refinement process. As a strategy to avoid this, we take the opportunity to use the *P*(model) factor in equation (11) to express the prior belief that the scattering curves are smooth as a function of energy, thus imposing restraints on Δ*f*′ and Δ*f*′′ for each metal atom and at each energy step. To cast these model parameters in terms of prior probability, we took the scattering curves for Fe^2+^ and Fe^3+^ in Fig. 1 ▸ as a reference distribution. In Fig. 1 ▸, the change in scattering factor with respect to energy has an approximately normal (Gaussian) distribution, with mean μ = 0.0 and standard deviation σ_1_ = 0.1 eV^−1^, while ΔΔ*f*′′/Δ*E* gives σ_2_ = 0.2 eV^−1^. Therefore, we express the overall prior probability of the model as a product of probabilities over both metal sites *m*, over *n* = 100 independent energy steps in the 7070–7170 eV range, and over both the dispersive and absorbtive corrections,
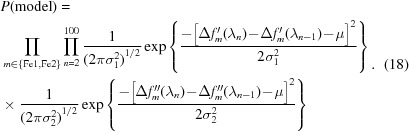
A corresponding term is incorporated into the loss function of equation (13),
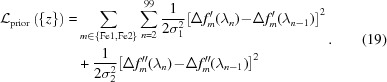
Scattering factors at *n* = 1 and *n* = 100 were not refined, thus constraining the values at 7071 and 7170 eV to their starting estimates.

#### 2.4.3. Model optimization   

Iterative parameter estimation was performed with the limited-memory Broyden–Fletcher–Goldfarb–Shanno (LBFGS) algorithm (Liu & Nocedal, 1989 ▸) as implemented in *CCTBX*. Initial estimates for the background parameters {*a_x_*,*b_x_*,*c_x_*} for each Bragg spot were obtained by masking out the Bragg signal (with a pixel mask determined by *DIALS*) and modeling the peripheral shoebox pixels only; however, subsequent iterations considered the entire shoebox when refining the background model. Requisite first derivatives are listed in the Appendices.[App appa]

#### 2.4.4. Implementation   

Table 2 ▸ lists the computational resources used for data analysis. Parallel execution with Python-mediated MPI was critical for keeping run times to within 30 h. However, work parcels were distributed in distinct patterns for various steps. The geometrical profiles ΔΩ_*L,i*_∑_*D*†_ **F** ^2^_latt_[**S**(λ)] depend primarily on the crystal orientation A* and the parameters {*a_x_*, *b_x_*, *c_x_*, *G_L_*}, but only weakly on the anomalous scattering factors Δ*f*′ and Δ*f*′′. The profiles are therefore pre-refined as step (*d*), which also happens to be the most computation-intensive step, while also refining {A*, *a_x_*, *b_x_*, *c_x_*, *G_L_*}, after which the geometric profiles are fixed. We then perform repeated macrocycles of step (*e*), refining {*a_x_*, *b_x_*, *c_x_*, *G_L_*}, and step (*f*), refining {Δ*f*′_*m*_(λ), Δ*f*′′_*m*_(λ)}. Although all the refineable parameters of steps (*e*)–(*f*) are, in principle, interdependent, and thus subject to simultaneous optimization, as a practical matter it is easier to refine the two parameter sets alternately until convergence is achieved. The anomalous correction refinement step (*f*) in particular has a complex implementation with respect to parallel execution. At each iteration within LBFGS, the structure factors |**F**_sim_(λ)|^2^ are initially calculated in MPI rank 0 and broadcast to all ranks. Individual ranks then calculate the separate contributions to 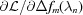 from various diffraction images, which are finally summed up by MPI.reduce() and are thereby available to rank 0 for the line search. In this programming pattern, the contributions of the restraints are handled by rank 0.

## 3. Results   

A total of 100 000 simulated diffraction patterns were processed with *dials.stills_process* (Method 3, Fig. 6 ▸). The 67 936 patterns with ≥3 *DIALS*-identified Bragg spots in the region of interest (Fig. 4 ▸) yielded 305 777 ‘shoeboxes’ (rectangular boxes each containing a Bragg spot plus background, Fig. 8 ▸), representing 100% of the 8241 unique Miller indices in the *C*2 asymmetric unit that span the 2.1–2.5 Å resolution range, implying an average 37-fold multiplicity of observation. These contained a total of 106 628 830 pixels (both background and Bragg spot) to be used for maximum-likelihood estimation of the energy-dependent anomalous scattering parameters at the two iron centers in ferredoxin.

**Figure 9:**
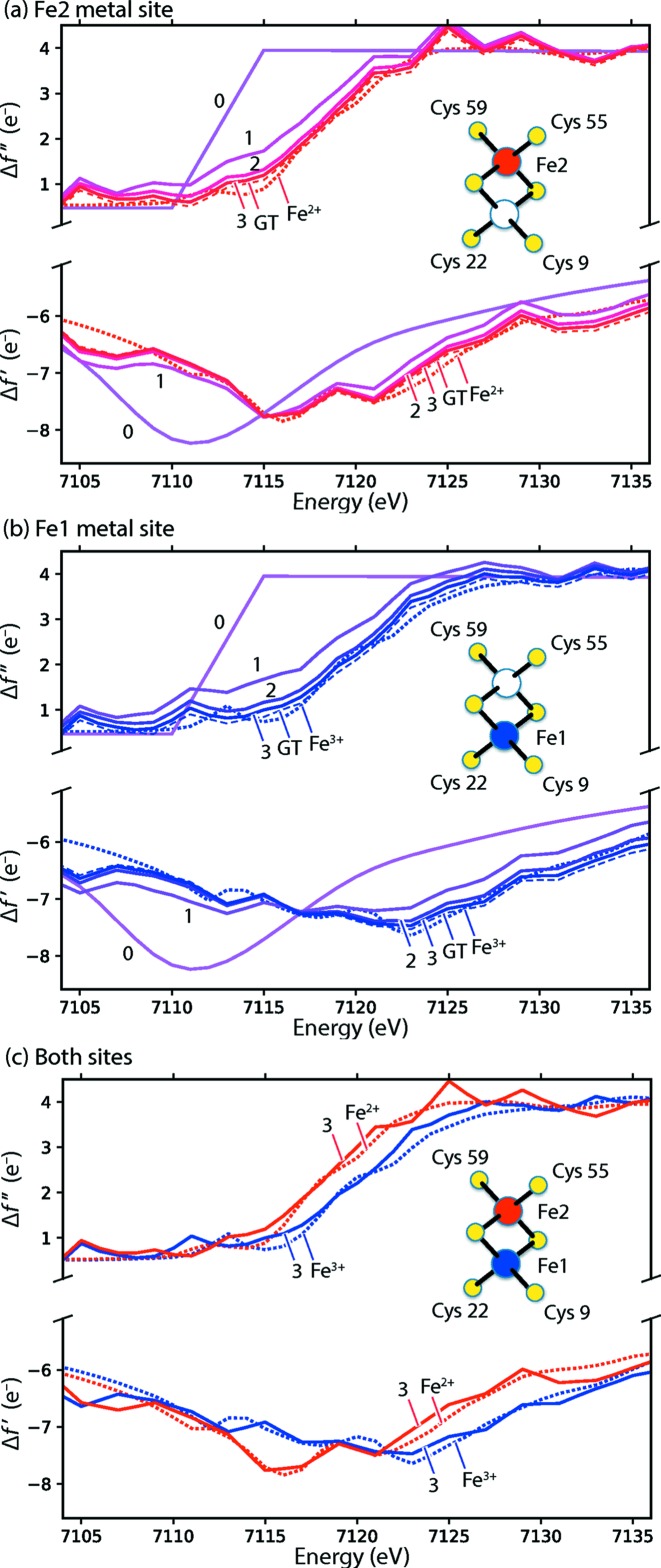
Anomalous scattering curves for the two iron centers converge to the ground truth. Progression of scattering factor parameter estimation is shown for metal sites (*a*) Fe2 and (*b*) Fe1. For each site, the starting values (0) are chosen to represent neutral metal iron atoms (Fe^0^), but after 1, 2 or 3 macrocycles the parameter estimates move stepwise closer to the true values (dotted lines) originating from Fe^2+^ or Fe^3+^ for (*a*) and (*b*), respectively. For comparison (thin dashed lines), the 3-macrocycle result is shown from a starting model representing the ground truth scattering factors (GT: Fe2 = Fe^2+^, Fe1 = Fe^3+^). (*c*) Direct comparison of both sites, showing that the oxidation state difference between Fe^2+^ and Fe^3+^ is clearly revealed by the refined 3-macrocycle models.

LBFGS parameter optimizations are summarized in Table 3 ▸, highlighting various starting models and conditions. For ease of comparison among many trials, Table 3 ▸ reports the root-mean-squared deviation of model scattering factors *versus* ground truth. Progress is best visualized (Fig. 9 ▸) by plotting the energy-dependence of the anomalous scattering factors {Δ*f*′_*m*_(λ), Δ*f*′′_*m*_(λ)}.

Of key interest is whether the inferred scattering curves can be used to distinguish valence states. Scattering from reduced (Fe^2+^) and oxidized (Fe^3+^) states is expected to differ in several regards (Einsle *et al.*, 2007 ▸; Sherrell, 2014 ▸; Fig. 1 ▸): the absorption *K*-edge [as shown by Δ*f*′′_*m*_(λ)] shifts 1–2 eV to a higher energy for the oxidized state, over roughly the 7115–7125 eV window, and corresponding changes are also seen in the dispersion spectrum [Δ*f*′_*m*_(λ)] at the pre-edge (7117 eV) and peak (7122–7132 eV) windows.

Table 3 ▸ indicates that the correct valence configuration is indeed readily determined by the analysis of our simulated data. We performed five parameter estimations, four of which started with guesses that incorrectly assign the valence state. One differed by switching the electron to the wrong Fe site, two by either the overall loss or the gain of one electron, and one involved the gain of five electrons (modeling the iron centers as metallic Fe^0^, which gives a very poor r.m.s.d. comparison of the ground truth). In all cases, including the use of the ground truth as the starting guess, the model refined to a state with a high degree of similarity to the ground truth.

These results suggest that our approach to parameter estimation is well behaved. Various starting guesses for the scattering factors yield essentially the same result, showing that we are comfortably within the radius of convergence (restraints described in Section 2.4.2 are necessary; data not shown). Convergence was achieved using a cohort of 50 000 input diffraction images. Utilizing a different cohort of 50 000 produces very similar agreement to ground truth. However, taking progressively smaller subsets degrades the performance, such that results from fewer than 25 000 images would be suspect. Shortcuts that involve fewer than three macrocycles (Figs. 5 ▸ and 9 ▸) would also be inadvisable as the interdependent treatments of {*a_x_*, *b_x_*, *c_x_*, *G_L_*} and {Δ*f*′_*m*_(λ), Δ*f*′′_*m*_(λ)} would have insufficient opportunity to cross-refine. Finally, we performed an important negative control: analysis with **h** = [*H*, *K*, *L*] replaced by [*H*, *K*, *L* + 1] in equation (1), leading to completely wrong scattering factors as expected.

To summarize, our simulation of XFEL diffraction patterns from a homogeneous and isomorphous population of 4 µm ferredoxin crystals with well characterized mosaicity shows that the pixel-profile analysis of Bragg spots in a small region of interest centered at 2.3 Å (from 50 000 patterns) determines the anomalous corrections Δ*f*′(λ) and Δ*f*′′(λ) for each of the two Fe atoms with sufficient precision to distinguish between the ferrous and ferric oxidation states. The calculation assumes an air path of 10 mm, a water path of 100 µm, and neglects diffuse scattering and absorption. It is assumed that the upstream single-shot spectrometer provides a good estimate of the incident spectra at the sample. It is also assumed that the unit-cell parameters are identical over the crystal population.^6^

## 4. Discussion   

The maximum-likelihood analysis presented above offers a path for using XFEL diffraction as a spatially resolved spectroscopic method. Anomalous scattering has the potential for distinguishing the electronic environment at metalloprotein metal sites (Einsle *et al.*, 2007 ▸), but such a measurement has yet to be achieved under the time-resolved, physiologically relevant conditions that are possible with XFELs. Several-atom cofactors such as the [4Mn:5O:Ca] oxygen-evolving complex of photosystem II have been investigated using X-ray emission spectroscopy at the *K*-edge, but this does not distinguish among the multiple Mn sites (Kern *et al.*, 2018 ▸). There are certainly many practical challenges: the anomalous scattering contribution is small compared with the overall diffraction (Fig. 7 ▸), the XFEL pulse’s broad bandpass smears out the energy-dependence of the signal (Figs. 1 ▸ and 8 ▸), and it has been notoriously difficult to scale XFEL-measured Bragg spots into self-consistent structure factor amplitudes. However, the consideration of simulated data (Table 3 ▸; Fig. 9 ▸) suggests that the anomalous scattering technique is possible with present XFEL instrumentation, provided that the incident X-ray spectra are measured to normalize the energy dependence (Zhu *et al.*, 2012 ▸; Fig. 3 ▸), the high-resolution Bragg diffraction is imaged by a pixel array positioned far enough back to spread out the energies (Fig. 8 ▸) and detailed physical modeling (such as *nanoBragg*) is applied to the signals from each pixel using sufficiently large datasets that are best analyzed by current petascale supercomputers.

The main result, the possibility of distinguishing valence states, rests on the relevance of the conditions we chose for the data simulation. We made every attempt to pick conservative parameters describing the crystal size and mosaicity, the liquid and air paths, the X-ray spectrum and intensity, and the solid angle subtended by the pixel array. Although early generation XFEL imaging detectors may have lacked the large dynamic range, linear response and well characterized gain needed to achieve our goals, we estimate that current-generation devices such as the ePix (Sikorski *et al.*, 2016 ▸), Jungfrau (Leonarski *et al.*, 2018 ▸) and AGIPD (Allahgholi *et al.*, 2015 ▸) offer the level of measurement stability that is incorporated into our assumptions. As for data analysis, it was important to model the contributions to each pixel from distributions of mosaic rotations and beam energies, the so-called ‘ray-tracing’ approach, and to properly weight the shot-noise statistical probability of each pixel value with equation (15). We note that an alternate calculation using only single-energy assignments for each pixel, and using an equally weighted non-linear least squares pixel treatment, failed to stably refine the anomalous corrections (data not shown). Translating our simulation into a real experiment will inevitably present additional systematic corrections such as proper calibration of the incident X-ray spectrometer, parallax effects in the imaging detector (Holton *et al.*, 2014 ▸; Winter *et al.*, 2018 ▸) and treatment of unexpectedly complex mosaic texture. Uncertainty in quantities such as the structure-factor phase angle from non-metals (in **F**_fixed_) may have to be integrated out (McCoy, 2004 ▸).

There may be additional scientific potential beyond what is anticipated in our simulation. Although most protein crystallography literature treats the anomalous corrections Δ*f*′ and Δ*f*′′ as scalar quantities, anisotropy has been reported in some cases, such that the scattering factor is represented by a tensor quantity, reflecting the complex chemical environment of the absorbing atom (Hendrickson *et al.*, 1988 ▸; Schiltz & Bricogne, 2008 ▸, 2010 ▸). In XFEL diffraction the crystals are examined in random orientations with respect to the polarized X-rays, which may thus offer the unique opportunity to sample the full rotational variation of the scattering, yielding additional details of the chemical environment.

Apart from its usefulness in modeling the anomalous scattering, detailed physical modeling as described above might play a future role in general XFEL data processing. Current XFEL data integration programs (White *et al.*, 2012 ▸, 2016 ▸; Kabsch, 2014 ▸; Brewster *et al.*, 2018 ▸) rely on pixel summation to obtain the signal intensity for each Bragg spot. In contrast, for synchrotron-based experiments that involve goniometer rotation, an alternate and more accurate method has long been available based on profile fitting. This has been achieved because there are standard theoretical frameworks for profile prediction (Otwinowski & Minor; 1997 ▸; Kabsch, 2010 ▸). Profile prediction has been discussed for XFEL work (Kroon-Batenburg *et al.*, 2015 ▸; White, 2014 ▸; Ginn *et al.*, 2015 ▸) but has not been widely applied. Fig. 8 ▸ illustrates the potential for quantitative description, showing that (i) each pixel of the Bragg spot represents a different average photon energy, (ii) each energy channel contributes to a narrow band of pixels, with adjacent-channel bands overlapping, and (iii) different energies contribute unequally to different spots, depending on how far (Δ**x**) the reciprocal lattice point is from the energy-specific Ewald sphere. Incorporating *nanoBragg* profile predictions into a data processing workflow such as *dials.stills_process* would provide a means for normalizing the Bragg spot intensities against the stochastically shaped incident spectra that can be measured for each pulse.

In a related matter it is interesting to speculate on what role the *nanoBragg* approach might play in optimizing the model parameters describing the crystal (the unit-cell parameters, orientation and mosaic texture). Two types of objective function have recently played a role in XFEL data modeling: the agreement of observed and predicted spot positions, and the agreement of observed and predicted spot intensities (post-refinement). Our results with positional refinement (using *dials.stills_process*, Fig. 6 ▸) illustrate that centroid spot positions do not give parameters such as crystal orientation to high accuracy. In post-refinement, the parameters are further refined to achieve the best intensity agreement among duplicate Miller index measurements after scaling for spot ‘partiality’, essentially the falloff of spot intensity as Δ**x** increases. Many post-refinement approaches have been explored for XFEL data (White, 2014 ▸; Kabsch, 2014 ▸; Sauter, 2015 ▸; Uervirojnangkoorn *et al.*, 2015 ▸; Ginn *et al.*, 2015 ▸; Kroon-Batenburg *et al.*, 2015 ▸). However, none of these were considered for use in this paper since the pixel summation step fundamentally erases the energy-dependent information that we sought to extract. Our approach represents a third type of objective function [equation (15)][Disp-formula fd15], which takes account of the nuanced spot sizes, shapes, and intensity profiles that are accessible when the analysis is done on a pixel-to-pixel basis. Indeed, spots that overlap for other reasons, such as multiple lattices or non-merohedral twins could be deconvoluted in this way. The material presented here provides a basic framework, and our initial results indicate that it is possible to refine crystal orientation to high accuracy (Fig. 6 ▸), at least with simulated data. The details of how to transfer these ideas to real experimental data remain to be worked out.

## 5. Conclusions   

The availability of XFEL beamlines has facilitated the study of proteins under physiological conditions free from radiation damage. For metalloenzymes in particular, time resolution has also been key for the study of catalytic mechanisms. In order to fully exploit the potential of time-resolved measurements, we have previously developed multimessenger techniques, simultaneously combining the results from X-ray diffraction for reporting the atomic structure, and X-ray emission spectroscopy for reporting the electronic state of active site transition metals (Kern *et al.*, 2013 ▸, 2018 ▸; Young *et al.*, 2016 ▸; Fuller *et al.*, 2017 ▸; Fransson *et al.*, 2018 ▸). Now, based on the current results, there is the potential of adding a third reporter to follow the time-dependence of the spatially resolved anomalous scattering factors and the underlying metal chemistry over the course of the reaction cycle. This information can be obtained without additional experiments, provided that the X-ray diffraction is collected at the metal absorption edge, hence avoiding the problems of normalization and comparability between different separate measurements. We hope that this approach will be a driver for future experimental design, and with respect to detectors and beam spectrometers, for XFEL endstation development.

## 6. Software availability   

The program *nanoBragg* is available as a standalone C program at https://bl831.als.lbl.gov/~jamesh/nanoBragg/. In this work, *nanoBragg* was ported into the open-source Python/C++ framework of *CCTBX* and can be downloaded at https://github.com/cctbx/cctbx_project. All scripts for reproducing this work are at https://github.com/nksauter/LS49, and in particular see the README file under paper1.

## Figures and Tables

**Table 1:** Supercomputing performance for the calculation of 100 000 diffraction images

Host	Intel architecture	Nodes requested (5% of each system)	CPU cores/node	Hardware threads/node	OpenMP threads/MPI rank	Total MPI ranks	Total wall time (h)	Image time (rank-sec)
edison.nersc.gov	Ivy Bridge	280	24	48	2	6720	12.3	2865
cori.nersc.gov	Haswell	120	32	64	2	3840	15.5	1855
cori.nersc.gov	Knights Landing	484	68	272	16	8228	7.2	1948

**Table 2:** Parallel execution of the data analysis

Processing step(s)	Work distributed to MPI ranks	Host and architecture	Nodes employed	CPU cores/node	Total MPI ranks	Total wall time (h)
(*a*)–(*c*) Spotfinding, indexing, refinement and integration with *DIALS*	Conventional data reduction on independent images	Linux server, AMD Opteron 6300	1	64	64	5.5
(*d*) Energy-dependent geometrical profile modeling for each spot, and profile-based refinement of crystal orientation	Each image processed independently	cori.nersc.gov, Knights Landing	400	68	6800	30.0
(*e*) Refinement of background and scale parameters	Each image processed independently	cori.nersc.gov, Knights Landing	32	68	1088	8.9
(*f*) Refinement of the scattering factors	Sum the independent contributions from each spot for each iteration					

**Table 3:** Maximum likelihood inference of spatially resolved anomalous scattering factors for the ferredoxin simulation Root-mean-squared agreement between the model and the ground truth anomalous scattering parameters were calculated over the 7105–7136 eV range. The number of crystal lattices used for parameter modeling was always less than the number of diffraction patterns selected for analysis due to the rejection of those images with two or fewer indexed shoeboxes in the Fig. 4 ▸ region of interest. Anomalous scattering factor refinements (24 LBFGS iterations per macrocycle, except the negative control, which used 12) were performed using the crystal rotation model from Method 4, while the spot background level and image scale factors were refined once per macrocycle.

	Starting valence state model for the two metal sites				R.m.s. agreement between model and ground truth scattering factors, including both metal sites *m* (e^−^)			
					Starting model		Refined model	
Comment	Fe1	Fe2	Number of macrocycles	Number of diffraction patterns (lattice models)	Δ*f*′_*m*_(λ)	Δ*f*′′_*m*_(λ)	Δ*f*′_*m*_(λ)	Δ*f*′′_*m*_(λ)
Negative control (*H*,*K*,*L* + 1)	+3	+2	1	50000 (33923)	0.0	0.0	7.786	5.673
Ground truth	+3	+2	3	50000 (33923)	0.0	0.0	0.180†	0.165†
Differing valence models as starting guess	+2	+2	3	50000 (33923)	0.265	0.278	0.182	0.167
	+2	+3	3	50000 (33923)	0.374	0.393	0.183	0.168
	+3	+3	3	50000 (33923)	0.265	0.278	0.184	0.164
	0	0	3	50000 (33923)	0.898†	1.235†	0.179†	0.198†
Fewer macrocycles	0	0	2	50000 (33923)			0.199†	0.281†
	0	0	1	50000 (33923)			0.332†	0.514†
Fewer images	0	0	3	1500 (1028)			0.327	0.306
	0	0	3	3000 (2051)			0.297	0.252
	0	0	3	6000 (4097)			0.291	0.246
	0	0	3	12000 (8181)			0.237	0.224
	0	0	3	25000 (17004)			0.191	0.210
Alternate (disjointed) data cohort	0	0	3	50000 (34012)			0.176	0.184

† Values correspond to the data shown in Fig. 9 ▸.

## Footnotes

1 With a 2 µm (FWHM) focus at the Linac Coherent Light Source, an X-ray fluence of 10^12^ photons per 50 fs pulse at 9.5 keV produces no photoreduction in Mn_2_^III, IV^O_2_-terpyridine (Alonso-Mori *et al.*, 2012 ▸).

2 One of the earliest demonstrations of anomalous scattering [Coster *et al.*, (1930 ▸); see also James (1962 ▸), p. 33]; involved the 85 eV splitting of the gold Lα doublet to either side of the crystal’s Zn edge. See also Arndt *et al.* (1982 ▸).

3 Other XFEL experiments have used back-position detectors to magnify the diffraction pattern (Chapman *et al.*, 2011 ▸; Brewster *et al.*, 2018 ▸; Duyvesteyn *et al.*, 2018 ▸).

4 Rather than treating the detector offset as a software issue to be recoded, we treated it as a beam position in need of precise refinement as discussed by Brewster *et al.* (2018 ▸). Briefly, the initial models from 2000 processed images were combined into a single file with *dials.combine_experiments*. The program *dials.refine* was then used to determine a single detector position consistent with all 2000 experiments.

5 In contrast to equation (6), where the simulated units are completely defined, equation (16) is intended to model actual experimental data. In this case, the absolute scale of several factors is unknown, including the number of incident photons *J*_0_(λ) measured on the X-ray spectrometer and the number of diffracting unit cells that enters into the **F** ^2^_latt_ factor.

6 In a real experiment, the true parameter distributions would need to have standard deviations of ≤0.02% in order to produce Bragg streaks, illustrated in Fig. 8 ▸(*a*), that are aligned along the energy axis to within ∼1 eV.

## Appendix A Derivatives for the image scale factor, the spot background and the anomalous corrections.

At each minimization step, LBFGS requires the first derivative of  with respect to each parameter *z*. With respect to the likelihood term

Fig. 5 ▸(*e*) describes the refinement of the overall scale factor for each image , and the background plane parameters  for each Bragg spot. Considering each parameter in turn,

where the pixel index *i* is over all Bragg spots of interest on a single image *L*, and

where the pixel index *i* spans a single Bragg spot *x*. Fig. 5 ▸(*f*) mentions the refinement of the anomalous corrections of metal *m* at wavelength λ,

where Δ*f_m_*(λ) is either Δ*f′_m_*(λ) or Δ*f′′_m_*(λ), and *I*_sim_(λ) is the structure factor intensity at the Miller index **h**_0_ associated with pixel *i* [computed as the dot product **F**_sim_(λ)·**F**_sim_(λ)]. Therefore,

Also, since the complex structure factor is a sum of individual atom contributions, we only need to consider the *N_M_* atoms within the class *m*,

and finally for the *s*th atom,

For the restraints expressed in the prior probability term, derivatives for the *n*th value of λ are

and

for *n* = 2, …, 99.

## Appendix B Derivatives for the crystal orientation

As mentioned in Section 2.3.2, the crystal orientation **A*** derived from *DIALS* (Method 3) is insufficient to model a spot profile close enough to that observed. Instead, it was necessary to produce an orientation that was modified (refined) by slight rotational perturbations,

where **R**_H_ and **R**_V_ are matrices that encode rotation about horizontal and vertical axes (perpendicular to the X-ray beam). The rotation values φ_H_ and φ_V_ are small, on the order of ±0.01°. To derive optimal values for each crystal [Fig. 5 ▸(*d*)] we use LBFGS parameter fitting, requiring the first derivative of  with respect to φ_H_ and φ_V_; these are refined while also refining scale factor *G_L_* for each image and background plane parameters *a_x_*, *b_x_*, *c_x_* for each Bragg spot in the framework of equations (15)–(17). However, in contrast to Appendix *A* where analytical derivatives are calculated for all other parameters (in order to determine the step gradient), we did not use an analytical form for the derivatives of equation (16) with respect to φ_H,V_. Although such an exercise is possible given the material presented above, it was expedient simply to use finite difference derivatives,

where φ_c_ is the current value of  at any given step of parameter refinement.
